# A Modular Grad-Div Stabilization Method for Time-Dependent Thermally Coupled MHD Equations

**DOI:** 10.3390/e24101336

**Published:** 2022-09-22

**Authors:** Xianzhu Li, Haiyan Su

**Affiliations:** College of Mathematics and System Science, Xinjiang University, Urumqi 830017, China

**Keywords:** thermally coupled MHD, modular grad-div stabilization, stability, optimal convergence

## Abstract

In this paper, we consider a fully discrete modular grad-div stabilization algorithm for time-dependent thermally coupled magnetohydrodynamic (MHD) equations. The main idea of the proposed algorithm is to add an extra minimally intrusive module to penalize the divergence errors of velocity and improve the computational efficiency for increasing values of the Reynolds number and grad-div stabilization parameters. In addition, we provide the unconditional stability and optimal convergence analysis of this algorithm. Finally, several numerical experiments are performed and further indicated these advantages over the algorithm without grad-div stabilization.

## 1. Introduction

The incompressible magnetohydrodynamic (MHD) model has a wide range of applications in scientific and engineering, such as electromagnetic pumping, liquid metal, electrolyte, and so on (see [1,2,3,4]). As we know, the  MHD model describes the interaction of an incompressible viscous conducting fluid and the electromagnetic field. In other words, it is a multi-physics phenomenon: the magnetic field changes the momentum of the fluid through the Lorenz force, and conversely, the conducting fluid influences the magnetic field through electric currents. When the buoyancy effects cannot be neglected in the momentum equation (owing to temperature differences in the flow), the MHD equations are usually coupled to the heat equation.

In this work, we consider the non-dimensional thermally coupled MHD equations with Boussinesq approximation as follows [5]:

(1) $$\left\{\begin{array}{c} u_{t}-\nu \Delta u+\left(u\cdot \nabla \right)u+\nabla p+SB\times curlB=\beta \theta +f_{1},\\ \nabla \cdot u=0,\\ B_{t}+R_{m}^{-1}\mathrm{curl}\mathrm{curl}B-\mathrm{curl}\left(u\times B\right)=0,\\ \nabla \cdot B=0,\\ \theta_{t}-\kappa \Delta \theta +\left(u\cdot \nabla \right)\theta =f_{2}, \end{array}\right.$$

which hold for all (x,t)∈Ω×[0,T], where T>0 is a given finite final time and $\Omega \subset R^{d}\left(d=2,3\right)$ is a bounded domain, u,B,p,θ represent the fluid velocity, magnetic field, pressure and temperature, respectively; $f_{1}$ and $f_{2}$ denote the external force terms. Some non-dimensional numbers in the above equations are characterized as follows:

$$\begin{array}{c} \alpha :=LU,\;\mathrm{thermally}\;\mathrm{diffusivity},\\ \nu^{-1}=R_{e}:=\frac{\rho UL}{\eta },\;\mathrm{Reynolds}\;\mathrm{number},\\ S:=\frac{B^{2}}{\mu \rho U^{2}},\;\mathrm{coupling}\;\mathrm{coefficient},\\ \beta :=\frac{\rho g\delta \theta L^{3}}{\alpha \eta },\;\mathrm{thermal}\;\mathrm{expansion}\;\mathrm{coefficient},\\ R_{m}:=\mu \sigma LU,\;\mathrm{Magnetic}\;\mathrm{Reynolds}\;\mathrm{number},\\ \kappa :=\rho C_{r}LU,\;\mathrm{thermal}\;\mathrm{conductivity}. \end{array}$$

Here, *L* is a characteristic length of the problem, *U* represents a characteristic velocity, *B* is a characteristic magnetic field, ρ is the fluid density, η is denoted as the fluid viscosity, μ is the magnetic permittivity, g is the gravitational acceleration vector, σ is the electric conductivity, $C_{r}$ is the adiabatic coefficient, δθ represents a characteristic temperature difference, usually computed from temperature boundary values when they are not zero. The initial values and boundary conditions of the system (1) are set as follows:

$$\begin{array}{c} u\left(x,0\right)=u^{0},\;B\left(x,0\right)=B^{0},\;\theta \left(x,0\right)=\theta^{0},\;\forall x\in \Omega ,\\ u=0,\;B\cdot n=0,\;n\times curlB=0,\;\mathrm{on}\;\partial \Omega \times [0,T]. \end{array}$$

In the recent years, much effort has been spent on the development of some efficient numerical methods to investigate this problem. Meir considered the existence and uniqueness of solutions for the thermally coupled MHD flow in [6], and developed the Galerkin finite-element method (FEM). Moreover, optimal error analysis of the model was established in [7]. A stabilized finite-element method was proposed in [8]. Furthermore, a decoupled Crank–Nicolson time-stepping scheme and partitioned time-stepping scheme for the thermally coupled MHD system were considered in [9,10], respectively, and some meaningful stability and convergence results were presented. Yang and Zhang [11] gave the convergence and stability analysis of three iterative methods of the steady thermally coupled MHD equations. In addition, Ding et al. studied convergence analysis of the Crank–Nicolson-extrapolated fully discrete scheme [5] and gave a fully discrete Euler semi-implicit scheme with the magnetic equation approximated by N$\overset{´}{e}$d$\overset{´}{e}$lec edge elements to capture the physical solutions [12], respectively. In addition, the modified characteristics finite-element method and projection method have been proposed in [13,14]. The unconditional stability of the fully discrete scheme and the optimal second-order convergent accuracy in both time and spatial discretizations were proved in [15]. Moreover, a linear fully decoupled velocity correction method for the thermally coupled MHD model was studied in [16].

It is worth mentioning that classical conforming finite-element discretizations for incompressible flows relax the divergence constraint, and give only a relatively weak limit. Although this enables us to construct a stable discretization of the inf-sup condition, a  weak limit will lead to errors in the continuous pressure that depends on the Reynolds number and causes inaccurate computational solutions for many flow problems. In order to overcome this difficulty, a grad-div stabilization was discovered for the first time in [17], which is a simple and popular method for improving mass conservation of numerical solutions and only adds a term that equals zero in a continuous equation. The analysis of grad-div stabilization for Stokes equations and Navier–Stokes equations were proposed in [18,19]. For the time-dependent Stokes/Darcy model, two grad-div stabilization methods were proposed in [20]. In addition, a grad-div stabilized projection finite-element method for a double-diffusive natural convection model was given in [21]. In view of this, a great deal of related interesting works have been reported in the recent years [22,23,24]. Although it is easy to implement it in the program, it also has some shortcomings: on the one hand, the stabilization makes it too difficult to solve due to an increased coupling in the system. On the other hand, too large values of grad-div parameters cause a low condition number of the corresponding linear system [25].

To solve the above problems, recently, a modular grad-div stabilization method has been proposed in [26], which allows the Navier–Stokes equations to be solved in two steps. Then, they also gave a BDF2 modular grad-div stabilization method for the Navier–Stokes equations in [27]. Next, the modular grad-div stabilization method of MHD and Boussinesq equations was proposed in [28,29], respectively. In [30], Li et al. presented a rotational pressure-correction method for the Stokes/Darcy model based on the modular grad-div stabilization.

As we know, the conservation of mass plays an important role in the construction of numerical schemes for incompressible fluids. Under certain extreme situations, nonphysical phenomena may appear if discrete solutions are not mass-conservative (see [31] for comprehensive discussions). Moreover, a large Reynolds number will cause the problem of convection dominance, which makes it very difficult to solve. Recently, many researchers are interested in studying highly efficient numerical algorithms for the thermally coupled MHD problem, but there is less attention to deal with the conservation of mass and high Reynolds number simultaneously. Since the modular grad-div stabilization not only improves the conservation of mass, but  has been proven useful for increasing values of the Reynolds number and grad-div parameters, the purpose of this paper is to apply the idea from [27] to the thermally coupled MHD model to improve the mass conservation of numerical solutions, and guarantee that the proposed algorithm is still effective for large Reynolds numbers. Here, we propose a first-order fully discrete modular grad-div stabilization method for the thermally coupled MHD equations, which adopts an Euler semi-implicit scheme for the time-discretization. This scheme is divided into two steps; in the first step, the intermediate velocity and other physical unknown quantities are solved. In the second step, we add two penalty terms to enforce improving the mass conservation and ensure high efficiency of the algorithm with large Reynolds numbers and grad-div stabilization parameters. Moreover, the unconditional stability and error estimation corresponding to this scheme are completed in this paper. Numerical examples further verify the reliability of the proposed algorithm.

This paper is arranged as follows. Section 2 describes the necessary notations and mathematical preliminaries. In Section 3, a fully-discrete modular grad-div stabilization method for the incompressible thermally coupled MHD equation is presented. In Section 4 and Section 5, we give its complete stability and convergence analysis, respectively. We also present some numerical experiments to illustrate the reliability and effectiveness of the method in Section 6. Finally, the last section summarizes the results of the paper.

## 2. Preliminaries

In this section, we use ${∥\cdot ∥}_{0,2}$ and (·,·) to denote the usual $L^{2}\left(\Omega \right)$ and its inner product. For $m\in N^{+},1\leq p\leq \infty ,$ the $L^{p}\left(\Omega \right)$ norm and $W^{m,p}\left(\Omega \right)$ norm are denoted by ${∥\cdot ∥}_{0,p}$ and ${∥\cdot ∥}_{m,p}$, respectively. Particularly, $H^{m}\left(\Omega \right)$ represents the case of p=2. In addition, X is defined as a normed function space in Ω, $L^{p}\left(0,T;X\right)$ is the space of all functions defined on Ω×(0,T), and the norm is bounded

$${∥u∥}_{L^{p}\left(0,T;X\right)}:=\int_{0}^{T}{∥u∥}_{X}^{p}dx,1\leq p\leq \infty .$$

These notations of Lebesgue and Sobolev spaces are used throughout this paper. We consider the classical function spaces as follows:

$$\begin{array}{c} X:={\left(H_{0}^{1}\left(\Omega \right)\right)}^{d}=\left\{v\in {\left(H^{1}\left(\Omega \right)\right)}^{d}:v=0\;\mathrm{on}\;\partial \Omega \right\},\\ Q:=L_{0}^{2}\left(\Omega \right)=\left\{q\in L^{2}\left(\Omega \right):\int_{\Omega }qdx=0\right\},\\ M:={\left(H_{n}^{1}\left(\Omega \right)\right)}^{d}=\left\{B\in {\left(H^{1}\left(\Omega \right)\right)}^{d}:B\cdot n=0\;\mathrm{on}\;\partial \Omega \right\},\\ W:=H_{0}^{1}\left(\Omega \right), \end{array}$$

the divergence-free subspaces of X and M are defined by:

$$X_{0}:=\left\{v\in X:\mathrm{div}v=0\right\},\;M_{0}:=\left\{B\in M:\mathrm{div}B=0\right\}.$$

From [32], we have the following two formulas

(a×b)×c·d=(a×b)·(c×d)=−(a×b)·(d×c),

$$\int_{\Omega }\mathrm{curl}\Phi \cdot \Psi \mathrm{dx}=-\int_{\partial \Omega }\left(\Phi \times n\right)\cdot \Psi \mathrm{ds}+\int_{\Omega }\Phi \cdot \mathrm{curl}\Psi \mathrm{dx},$$

which imply that for all Φ, Ψ∈M and w∈X,

$$\left(\mathrm{curl}(w\times \Phi ),\Psi \right){=-<\left(w\times \Phi \right)\times n,\Psi >|}_{\partial \Omega }+\left(w\times \Phi ,\mathrm{curl}\Psi \right)=-\left(\mathrm{curl}\Psi \times \Phi ,w\right).$$

Therefore, the weak formulation of (1) reads: find $\left(u,p,B,\theta \right)\in L^{2}\left(0,T;X\right)\times L^{2}\left(0,T;Q\right)$×$L^{2}\left(0,T;M\right)\times L^{2}\left(0,T;W\right),$ such that for all (v,q,C,φ)∈X×Q×M×W,

(2) $$\left\{\begin{array}{c} \left(u_{t},v\right)+\nu \left(\nabla u,\nabla v\right)+b\left(u,u,v\right)+S\left(B\times curlB,v\right)-\left(p,\nabla \cdot v\right)+\left(\nabla \cdot u,q\right)\\ =(\beta \theta +f_{1},v),\\ \left(B_{t},C\right)+R_{m}^{-1}\left(\mathrm{curl}B,\mathrm{curl}C\right)-\left(B\times curlC,u\right)=0,\\ \left(\theta_{t},\varphi \right)+\kappa \left(\nabla \theta ,\nabla \varphi \right)+b\left(u,\theta ,\varphi \right)=\left(f_{2},\varphi \right),\\ u\left(x,0\right)=u^{0},\;B\left(x,0\right)=B^{0}, \end{array}\right.$$

with

$$\begin{array}{c} \;\;\;\;\;\;\;\;\;\;b\left(u,v,w\right)=\left(\left(u\cdot \nabla \right)v,w\right)+\frac{1}{2}\left(\left(\nabla \cdot u\right)w,v\right)=\frac{1}{2}\left(\left(u\cdot \nabla \right)v,w\right)-\frac{1}{2}\left(\left(u\cdot \nabla \right)w,v\right),\;\forall u,v,w\in X. \end{array}$$

Furthermore, the analysis of time-discretization utilizes the following norms, for 1≤m<∞:

$${\left|||v||\right|}_{m,\infty }:=\underset{1\leq n\leq N}{\sup}∥v^{n}{∥}_{m},\;|||v^{n}{\left||\right|}_{m,p}:=(\tau \sum_{n=0}^{N-1}∥v^{n}{{∥}_{m}^{p})}^{\frac{1}{p}}.$$

For u,v,w∈X, we have some properties of these trilinear forms (see [33])

(3) $$\begin{array}{c} \begin{array}{cc} & \left|b\left(u,v,w\right)\right|\leq C_{0}{∥\nabla u∥}_{0,2}{∥\nabla v∥}_{0,2}{∥\nabla w∥}_{0,2},\\ & \left|b\left(u,v,w\right)\right|\leq C_{1}{∥u∥}_{0,2}^{\frac{1}{2}}{∥\nabla u∥}_{0,2}^{\frac{1}{2}}{∥\nabla v∥}_{0,2}{∥\nabla w∥}_{0,2}. \end{array} \end{array}$$

Additionally, for v∈XandB,C∈M from [34,35], we have the following bounds:

(4) $$\begin{array}{c} \begin{array}{cc} \left|(v\times B,\mathrm{curl}C)\right| & \leq C_{3}{∥\nabla v∥}_{0,2}{∥\mathrm{curl}B∥}_{0,2}{∥\mathrm{curl}C∥}_{0,2},\\ \left|(v\times B,\mathrm{curl}C)\right| & \leq C_{4}{∥v∥}_{2,2}{∥B∥}_{0,2}{∥\mathrm{curl}C∥}_{0,2},\\ \left|(v\times B,\mathrm{curl}C)\right| & \leq C_{5}{∥\nabla v∥}_{0,2}{∥B∥}_{2,2}{∥C∥}_{0,2}. \end{array} \end{array}$$

Here and after, *C* (with or without a subscript) denotes a general positive constant, which may represent different values in different situations. In addition, we need the following assumptions on the prescribed data for problem (1), as it is useful for our later theoretical analysis.

**Assumption** **A1.** *The initial data $u^{0}\in X_{0}\cap H^{2}{\left(\Omega \right)}^{d},B^{0}\in M_{0}\cap H^{2}{\left(\Omega \right)}^{d},\theta^{0}\in W_{0}\cap H^{2}\left(\Omega \right),f_{1}$ and $f_{2}$ satisfy*

$$\begin{array}{c} ∥u^{0}{∥}_{2,2}+∥B^{0}{∥}_{2,2}+∥\theta^{0}{∥}_{2,2}+\underset{0\leq t\leq T}{\sup}(∥f_{1}{∥}_{0,2}+∥f_{1t}{∥}_{0,2}+∥f_{2}{∥}_{0,2}+∥f_{2t}{∥}_{0,2})\leq C. \end{array}$$

**Assumption** **A2.** *The problem (1) has a unique local strong solution (u,p,B,θ) on [0,T] such that*

$$\begin{array}{c} \underset{0\leq t\leq T}{\sup}{(∥u\left(t\right)∥}_{2,2}+{∥B\left(t\right)∥}_{2,2}+{∥\theta \left(t\right)∥}_{2,2}+{∥p\left(t\right)∥}_{1,2}+∥u_{t}{\left(t\right)∥}_{0,2}+∥B_{t}{\left(t\right)∥}_{0,2}+∥\theta_{t}\left(t\right){∥}_{0,2})\\ +\int_{0}^{T}(∥\nabla u_{t}{∥}_{0,2}^{2}+∥\nabla B_{t}{∥}_{0,2}^{2}+∥\nabla \theta_{t}{∥}_{0,2}^{2}+∥u_{tt}{∥}_{-1,2}^{2}+∥B_{tt}{∥}_{-1,2}^{2}+∥\theta_{tt}{∥}_{-1,2}^{2})\leq C. \end{array}$$

**Assumption** **A3.** *Assume that the boundary of* Ω *is smooth so that the unique solution (v,q) of the Stokes problem in [34]*

$$-\Delta u+\nabla q=f_{u}{,\mathrm{div}v=0\;\mathrm{in}\;\Omega ,\;\;v|}_{\partial \Omega }=0,$$

*for prescribed $f_{u}\in L^{2}{\left(\Omega \right)}^{d}$ satisfies*

$${∥v∥}_{2,2}+{∥q∥}_{1,2}\leq C{∥f_{u}∥}_{0,2},$$

*and Maxwell’s equations*

$$\begin{array}{cc} \; & \mathrm{curl}\mathrm{curl}B=f_{B},\mathrm{div}B=0\;\mathrm{in}\;\Omega ,\\ \; & n\times \mathrm{curl}B=0,\;B\cdot n=0\;on\;\partial \Omega , \end{array}$$

*for prescribed $f_{B}\in L^{2}{\left(\Omega \right)}^{d}$ admit a unique solution $B\in M_{0}$ which satisfies*

$${∥B∥}_{2,2}\leq C{∥f_{B}∥}_{0,2}.$$

The following lemma is very important in convergence analysis; so, we recall it from [34].

**Lemma** **1** (Discrete Gronwall’s Lemma). *Let $a_{n},b_{n}$ and $d_{n}$ for the integer n≥0 be nonnegative numbers such that*

$$\begin{array}{c} a_{m}+\tau \sum_{n=0}^{m}b_{n}\leq \tau \sum_{n=0}^{m-1}a_{n}d_{n}+C_{*},\;m\geq 1, \end{array}$$

*then*

$$\begin{array}{c} a_{m}+\tau \sum_{n=0}^{m}b_{n}\leq C_{*}\exp\left(\tau \sum_{n=0}^{m-1}d_{n}\right),\;m\geq 1. \end{array}$$

For the spatial discretization, we define the following finite spaces, where (u,p) using the finite-element pair $\left(P_{1}^{b},P_{1}\right)$ and B,θ using $P_{1}$ element. Let $\pi_{h}=\left\{K\right\}$ be a uniformly regular family of triangulation of Ω, and define the mesh size $h=\max_{K\in \pi_{h}}\left\{h_{K},h_{K}=diam\left(K\right)>0\right\}$.

$$\begin{array}{cc} \; & X_{h}:={\left(P_{1}^{b}\right)}^{d}\cap X,\\ \; & Q_{h}:=\left\{q_{h}\in C^{0}{\left(\Omega \right)}^{d}\cap Q:q_{h}{|}_{K}\in P_{1}\left(K\right),\forall K\in \pi_{h}\right\},\\ \; & M_{h}:=\left\{B_{h}\in C^{0}{\left(\Omega \right)}^{d}\cap M:B_{h}{|}_{K}\in P_{1}\left(K\right),\forall K\in \pi_{h}\right\},\\ \; & W_{h}:=\left\{\theta_{h}\in C^{0}{\left(\Omega \right)}^{d}\cap W:\theta_{h}{|}_{K}\in P_{1}\left(K\right),\forall K\in \pi_{h}\right\}. \end{array}$$

Here, $P_{1}^{b}$ is defined as (more details see [32])

$$P_{1}^{b}:=\left\{v_{h}\in C^{0}{\left(\Omega \right)}^{d}:v_{h}{|}_{K}\in P_{1}\left(K\right)⊕span\left\{\hat{b}\right\},\forall K\in \pi_{h}\right\}.$$

Furthermore, we need the subspace $X_{0h}$ of $X_{h}$ which is defined as

$$X_{0h}:=\left\{v_{h}\in X_{h}:\left(\nabla \cdot v_{h},q\right)=0,\forall q_{h}\in Q_{h}\right\}.$$

Let $\left(v,q\right)\in X_{0}\times Q$, we denote by $\left(R_{h}\left(v,q\right),q_{h}\left(v,q\right)\right)\in X_{h}\times Q_{h}$ the so-called Stokes projection. The projections satisfy the following properties (see [32]):

$$∥R_{h}{-v∥}_{0,2}+h(∥\nabla R_{h}{-v∥}_{0,2}+∥q_{h}{-q∥}_{0,2})\leq Ch^{2}{(∥Av∥}_{0,2}+{∥q∥}_{1,2}),$$

for all $\left(v,q\right)\in \left(H^{2}\cap X\right)\times \left(H^{1}\cap Q\right),$ where a discrete analogue $A_{h}=-P_{h}\Delta_{h}$ of the Stokes operator *A* is defined through the condition that $\left(-\Delta_{h}u_{h},v_{h}\right)=\left(\nabla u_{h},\nabla v_{h}\right)$ for all $u_{h},v_{h}\in X_{h}.$

Next, we give the numerical scheme of this paper.

## 3. A Modular Grad-Div Stabilization Method for Time-Dependent Thermally Coupled MHD Equations

  Now, we construct a fully-discrete numerical scheme for solving the model system (1) and prove the corresponding unconditional stability. Divide the simulation time *T* into *N* smaller time intervals $\left[0,T\right]={⋃}_{n=0}^{N-1}\left[t_{n},t_{n+1}\right]$, where $t_{n}=n\tau ,T=N\tau .$ Our numerical scheme reads as follows.

## 4. Stability Analysis

Now, we focus on the stability of Algorithm 1. Our stability analysis shows that approximate solutions of Algorithm 1 are stable without any time step restriction. In order to obtain the stability result, we first present a lemma which gives a relation between solutions of Step 1 and Step 2.
**Algorithm 1** A Modular Grad-Div Stabilization Method**Step 1**: For all $\left(v_{h},q_{h},C_{h},\varphi_{h}\right)\in \left(X_{h},Q_{h},M_{h},W_{h}\right)$, find $({\hat{u}}_{h}^{n+1},p_{h}^{n+1},B_{h}^{n+1},\theta_{h}^{n+1})\in$$\left(X_{h},Q_{h},M_{h},W_{h}\right)$ such that
(5)$$\left\{\begin{array}{c} \left(d_{t}\theta_{h}^{n+1},\varphi_{h}\right)+\kappa \left(\nabla \theta_{h}^{n+1},\nabla \varphi_{h}\right)+b\left(u_{h}^{n},\theta_{h}^{n+1},\varphi_{h}\right)=\left(f_{2}^{n+1},\varphi_{h}\right),\\ \left(d_{t}{\hat{u}}_{h}^{n+1},v_{h}\right)+\nu \left(\nabla {\hat{u}}_{h}^{n+1},\nabla v_{h}\right)+b\left(u_{h}^{n},{\hat{u}}_{h}^{n+1},v_{h}\right)-\left(p_{h}^{n+1},\nabla \cdot v_{h}\right)\\ +\left(\nabla \cdot {\hat{u}}_{h}^{n+1},q_{h}\right)+S\left(B_{h}^{n}\times \mathrm{curl}B_{h}^{n+1},v_{h}\right)=\left(\beta \theta_{h}^{n+1}+f_{1}^{n+1},v_{h}\right),\\ \left(d_{t}B_{h}^{n+1},C_{h}\right)+1/R_{m}\left(\mathrm{curl}B_{h}^{n+1},\mathrm{curl}C_{h}\right)-\left({\hat{u}}_{h}^{n+1}\times B_{h}^{n},\mathrm{curl}C_{h}\right)=0. \end{array}\right.$$**Step 2**: For all $v_{h}\in X_{h}$, find $u_{h}^{n+1}\in X_{h}$ such that
(6)$$\begin{array}{c} \left(\frac{u_{h}^{n+1}-{\hat{u}}_{h}^{n+1}}{\tau },v_{h}\right)+\beta_{0}\left(d_{t}\nabla \cdot u_{h}^{n+1},\nabla \cdot v_{h}^{n+1}\right)+\gamma_{0}\left(\nabla \cdot u_{h}^{n+1},\nabla \cdot v_{h}^{n+1}\right)=0, \end{array}$$
where $u_{h}^{0}={\hat{u}}_{h}^{0},$$d_{t}{\hat{u}}_{h}^{n+1}=\frac{1}{\tau }\left({\hat{u}}_{h}^{n+1}-u_{h}^{n}\right),$$d_{t}s_{h}^{n+1}=\frac{1}{\tau }\left(s_{h}^{n+1}-s_{h}^{n}\right),s=\theta$ or s=B, and the stabilization parameters $\beta_{0},\gamma_{0}\geq 0.$

**Lemma** **2.** *Let $u_{h}^{n+1}$ be solutions to (6). Then, it holds*

$$\begin{array}{cc} ∥{\hat{u}}_{h}^{n+1}{∥}_{0,2}^{2}= & ∥u_{h}^{n+1}{∥}_{0,2}^{2}+∥u_{h}^{n+1}-{\hat{u}}_{h}^{n+1}{∥}_{0,2}^{2}+2\gamma_{0}\tau {∥\nabla \cdot u_{h}^{n+1}∥}_{0,2}^{2}\\ \; & +\beta_{0}(∥\nabla \cdot u_{h}^{n+1}{∥}_{0,2}^{2}-∥\nabla \cdot u_{h}^{n}{∥}_{0,2}^{2}+∥\nabla \cdot \left(u_{h}^{n+1}-u_{h}^{n}\right){∥}_{0,2}^{2}). \end{array}$$

**Proof.**  We choose $v_{h}=2\tau u_{h}^{n+1}$ in (6) and use $2\left(a-b,a\right)={\left|a\right|}^{2}-{\left|b\right|}^{2}+{\left|a-b\right|}^{2}$, rearrange terms to obtain the desired estimates. □

**Theorem** **1.** *Assume that $f_{1},f_{2}\in L^{2}\left(0,T;H^{-1}\left(\Omega \right)\right)$. Then, the solutions of Algorithm 1 satisfy the following: for any τ>0, and we set $H=\kappa^{-1}\tau \sum_{n=0}^{N-1}∥f_{2}^{n+1}{∥}_{-1}^{2}+{∥\theta_{h}^{0}∥}_{0,2}^{2}.$*

$$\begin{array}{cc} \; & ∥u_{h}^{N}{∥}_{0,2}^{2}+S∥B_{h}^{N}{∥}_{0,2}^{2}+\beta_{0}∥\nabla \cdot u_{h}^{N}{∥}_{0,2}^{2}+∥\theta_{h}^{N}{∥}_{0,2}^{2}+\tau \sum_{n=0}^{N-1}(\nu ∥\nabla {\hat{u}}_{h}^{n+1}{∥}_{0,2}^{2}+2SR_{m}^{-1}{∥\nabla \times B_{h}^{n+1}∥}_{0,2}^{2}\\ & +2\gamma_{0}∥\nabla \cdot u_{h}^{n+1}{∥}_{0,2}^{2}+\kappa ∥\nabla \theta_{h}^{n+1}{∥}_{0,2}^{2})+\sum_{n=0}^{N-1}(∥{\hat{u}}_{h}^{n+1}-u_{h}^{n}{∥}_{0,2}^{2}+{∥u_{h}^{n+1}-{\hat{u}}_{h}^{n+1}∥}_{0,2}^{2}\\ \; & +\beta_{0}∥\nabla \cdot u_{h}^{n+1}-\nabla \cdot u_{h}^{n}{∥}_{0,2}^{2}+S∥B_{h}^{n+1}-B_{h}^{n}{∥}_{0,2}^{2}+∥\theta_{h}^{n+1}-\theta_{h}^{n}{∥}_{0,2}^{2})\\ \; & \leq ∥u_{h}^{0}{∥}_{0,2}^{2}+S∥B_{h}^{0}{∥}_{0,2}^{2}+\beta ∥\nabla \cdot u_{h}^{0}{∥}_{0,2}^{2}+{∥\theta_{h}^{0}∥}_{0,2}^{2}+2\nu^{-1}\beta^{2}C_{p}^{2}\kappa^{-1}H\\ \; & +\tau \sum_{n=0}^{N-1}(2\nu^{-1}∥f_{1}^{n+1}{∥}_{-1}^{2}+\kappa^{-1}∥f_{2}^{n+1}{∥}_{-1}^{2})\\ \; & \leq C, \end{array}$$

**Proof.**  We first prove the temperature stability result. We set $\varphi_{h}=2\tau \theta_{h}^{n+1}$ in (5), apply the Canchy–Schwarz and Young’s inequalities on the right hand side, yielding

(7) $$\begin{array}{c} ∥\theta_{h}^{n+1}{∥}_{0,2}^{2}-∥\theta_{h}^{n}{∥}_{0,2}^{2}+∥\theta_{h}^{n+1}-\theta_{h}^{n}{∥}_{0,2}^{2}+\kappa \tau ∥\nabla \theta_{h}^{n+1}{∥}_{0,2}^{2}\leq \kappa^{-1}\tau {∥f_{2}^{n+1}∥}_{-1}^{2}. \end{array}$$

For Equations (5), we set $v_{h}=2\tau {\hat{u}}_{h}^{n+1},C_{h}=2S\tau B_{h}^{n+1}$ to get

(8) $$\begin{array}{c} \begin{array}{c} ∥{\hat{u}}_{h}^{n+1}{∥}_{0,2}^{2}-∥u_{h}^{n}{∥}_{0,2}^{2}+∥{\hat{u}}_{h}^{n+1}-u_{h}^{n}{∥}_{0,2}^{2}+\nu \tau {∥\nabla {\hat{u}}_{h}^{n+1}∥}_{0,2}^{2}+2S\tau \left(B_{h}^{n}\times \mathrm{curl}B_{h}^{n+1},{\hat{u}}_{h}^{n+1}\right)\\ \leq 2\beta^{2}C_{p}^{4}\nu^{-1}\tau ∥\nabla \theta_{h}^{n+1}{∥}_{0,2}^{2}+2\nu^{-1}\tau {∥f_{1}^{n+1}∥}_{-1}^{2}. \end{array} \end{array}$$

$$\begin{array}{cc} S(∥B_{h}^{n+1}{∥}_{0,2}^{2}-∥B_{h}^{n}{∥}_{0,2}^{2}+∥B_{h}^{n+1}-B_{h}^{n}{∥}_{0,2}^{2}) & +2SR_{m}^{-1}\tau {∥\nabla \times B_{h}^{n+1}∥}_{0,2}^{2}\\ \; & -2S\tau ({\hat{u}}_{h}^{n+1}\times B_{h}^{n},\nabla \times B_{h}^{n+1})=0. \end{array}$$

From (7), we obtain

$$\tau \sum_{n=0}^{N-1}∥\theta^{n+1}{∥}_{0,2}^{2}\leq \kappa^{-1}(\kappa^{-1}\tau \sum_{n=0}^{N-1}∥f_{2}^{n+1}{∥}_{-1}^{2}+∥\theta^{0}{∥}_{0,2}^{2})=\kappa^{-1}H.$$

The obtained results are substituted into (8), then summed over time steps with this estimate, and finally, rearranging terms finishes the proof. □

## 5. Error Analysis

In this section, we show that solutions of the proposed algorithm converge to the true solutions of (1). In order to obtain the equations, we denote true solutions at time level $t_{n+1}$, the error analysis needs the following error decomposition at time level $t_{n+1}$:

$$\begin{array}{cc} {\hat{e}}_{u}^{n+1}: & =u\left(t_{n+1}\right)-{\hat{u}}_{h}^{n+1}=\left(u\left(t_{n+1}\right)-{\tilde{u}}^{n+1}\right)-\left({\hat{u}}_{h}^{n+1}-{\tilde{u}}^{n+1}\right):=\eta_{u}^{n+1}-\Lambda_{u,h}^{n+1},\\ e_{u}^{n+1}: & =u\left(t_{n+1}\right)-u_{h}^{n+1}=\left(u\left(t_{n+1}\right)-{\tilde{u}}^{n+1}\right)-\left(u_{h}^{n+1}-{\tilde{u}}^{n+1}\right):=\eta_{u}^{n+1}-\phi_{u,h}^{n+1},\\ e_{\theta }^{n+1}: & =\theta \left(t_{n+1}\right)-\theta_{h}^{n+1}=\left(\theta \left(t_{n+1}\right)-{\tilde{\theta }}^{n+1}\right)-\left(\theta_{h}^{n+1}-{\tilde{\theta }}^{n+1}\right):=\eta_{\theta }^{n+1}-\phi_{\theta ,h}^{n+1},\\ e_{B}^{n+1}: & =B\left(t_{n+1}\right)-B_{h}^{n+1}=\left(B\left(t_{n+1}\right)-{\tilde{B}}^{n+1}\right)-\left(B_{h}^{n+1}-{\tilde{B}}^{n+1}\right):=\eta_{B}^{n+1}-\phi_{B,h}^{n+1}, \end{array}$$

where ${\tilde{u}}^{n+1}$ denotes interpolation of $u(t_{n+1})$ in $X_{h}$, ${\tilde{\theta }}^{n+1}$ denotes interpolation of $\theta (t_{n+1})$ in $W_{h}$, ${\tilde{B}}^{n+1}$ denotes interpolation of $B(t_{n+1})$ in $M_{h}$.

**Lemma** **3.** *Consider the second step of Algorithm 1, then it holds (see [29])*

$$\begin{array}{cc} ∥\Lambda_{u,h}^{n+1}{∥}_{0,2}^{2} & \geq ∥\phi_{u,h}^{n+1}{∥}_{0,2}^{2}+∥\Lambda_{u,h}^{n+1}-\phi_{u,h}^{n+1}{∥}_{0,2}^{2}+\beta_{0}(∥\nabla \cdot \phi_{u,h}^{n+1}{∥}_{0,2}^{2}-∥\nabla \cdot \phi_{u,h}^{n}{∥}_{0,2}^{2})\\ \; & +\frac{\beta_{0}}{2}∥\nabla \cdot \left(\phi_{u,h}^{n+1}-\phi_{u,h}^{n}\right){∥}_{0,2}^{2}+\gamma_{0}\tau ∥\nabla \cdot \phi_{u,h}^{n+1}{∥}_{0,2}^{2}-\beta_{0}\tau {∥\nabla \cdot \phi_{u,h}^{n}∥}_{0,2}^{2}\\ \; & -\beta_{0}\left(1+2\tau \right)\int_{t_{n}}^{t_{n+1}}∥\nabla \eta_{u,t}{∥}_{0,2}^{2}dt-\gamma_{0}\tau {∥\nabla \eta_{u}^{n+1}∥}_{0,2}^{2}. \end{array}$$

**Theorem** **2.** *Suppose that Assumption 1–3 are satisfied, then the following estimate holds*

$$\begin{array}{cc} \; & ∥e_{u}^{N}{∥}_{0,2}^{2}+\beta_{0}∥\nabla \cdot e_{u}^{N}{∥}_{0,2}^{2}+S∥e_{B}^{N}{∥}_{0,2}^{2}+∥e_{\theta ,h}^{N}{∥}_{0,2}^{2})\\ \; & +\tau \sum_{n=0}^{N-1}(\nu ∥\nabla {\hat{e}}_{u}^{n+1}{∥}_{0,2}^{2}+\frac{S}{R_{m}}∥\mathrm{curl}e_{B}^{n+1}{∥}_{0,2}^{2}+\kappa ∥\nabla e_{\theta }{∥}_{0,2}^{2}+\gamma_{0}∥\nabla \cdot e_{u}^{n+1}{∥}_{0,2}^{2})\\ \; & \leq C(\tau h^{2}+\tau h+h^{2}+\tau^{2}). \end{array}$$

**Proof.**  For simplicity, our entire proof process is divided into three steps, as shown below.

**Step 1**: [**The derivation of error equations**] Let $\left(v,q\right)=\left(v_{h},q_{h}\right),C=C_{h},\varphi =\varphi_{h}$ in (2) with $t=t_{n+1}$, and use integration by parts to get

(9) $$\begin{array}{c} \left(d_{t}u\left(t_{n+1}\right),v_{h}\right)+a\left(u\left(t_{n+1}\right),v_{h}\right)+b\left(u\left(t_{n+1}\right),u\left(t_{n+1}\right),v_{h}\right)\\ -S\left(\mathrm{curl}B\left(t_{n+1}\right)\times B\left(t_{n+1}\right),v_{h}\right)-d\left(v_{h},p\left(t_{n+1}\right)\right)+d\left(u\left(t_{n+1}\right),q_{h}\right)\\ =\left(f_{1}\left(t_{n+1}\right),v_{h}\right)+\beta \left(\theta \left(t_{n+1}\right),v_{h}\right)-\frac{1}{\tau }\int_{t_{n}}^{t_{n+1}}\left(t-t_{n}\right)\left(u_{tt}\left(t\right),v_{h}\right)dt, \end{array}$$

(10) $$\begin{array}{c} \left(d_{t}B\left(t_{n+1}\right),C_{h}\right)+\frac{1}{R_{m}}\left(\mathrm{curl}B\left(t_{n+1}\right),\mathrm{curl}C_{h}\right)-\left(u\left(t_{n+1}\right)\times B\left(t_{n+1}\right),\mathrm{curl}C_{h}\right)\\ =-\frac{1}{\tau }\int_{t_{n}}^{t_{n+1}}\left(t-t_{n}\right)\left(B_{tt}\left(t\right),C_{h}\right)dt, \end{array}$$

(11) $$\begin{array}{c} \left(d_{t}\theta \left(t_{n+1}\right),\varphi_{h}\right)+\kappa \left(\nabla \theta \left(t_{n+1}\right),\nabla \varphi_{h}\right)+b\left(u\left(t_{n+1}\right),\theta \left(t_{n+1}\right),\varphi_{h}\right)=\left(f_{2}\left(t_{n+1}\right),\varphi_{h}\right). \end{array}$$

Then, subtracting (5) from (9)–(11), respectively, we obtain

(12) $$\begin{array}{c} \left(d_{t}{\hat{e}}_{u}^{n+1},v_{h}\right)+a\left({\hat{e}}_{u}^{n+1},v_{h}\right)+b\left(u\left(t_{n}\right),u\left(t_{n+1}\right),v_{h}\right)-b\left(u_{h}^{n},{\hat{u}}_{h}^{n+1},v_{h}\right)-d\left(v_{h},p\left(t_{n+1}\right)-p_{h}^{n+1}\right)\\ +d\left({\hat{e}}_{u}^{n+1},q_{h}\right)+S\left(B\left(t_{n}\right)\times \mathrm{curl}B\left(t_{n+1}\right),v_{h}\right)-S\left(B_{h}^{n}\times \mathrm{curl}B_{h}^{n+1},v_{h}\right)\\ =\beta \left(e_{\theta }^{n+1},v_{h}\right)+\left(E_{1}^{n+1},v_{h}\right), \end{array}$$

(13) $$\begin{array}{c} \left(d_{t}e_{B}^{n+1},C_{h}\right)+\frac{1}{R_{m}}\left(\mathrm{curl}e_{B}^{n+1},\mathrm{curl}C_{h}\right)-\left(u\left(t_{n+1}\right)\times B\left(t_{n}\right),\mathrm{curl}C_{h}\right)\\ +\left({\hat{u}}_{h}^{n+1}\times B_{h}^{n},\mathrm{curl}C_{h}\right)=\left(E_{2}^{n+1},C_{h}\right), \end{array}$$

(14) $$\begin{array}{c} \left(d_{t}e_{\theta }^{n+1},\varphi_{h}\right)+\kappa \left(\nabla e_{\theta }^{n+1},\nabla \varphi_{h}\right)+b\left(u\left(t_{n}\right),\theta \left(t_{n+1}\right),\varphi_{h}\right)-b\left(u_{h}^{n},\theta_{h}^{n+1},\varphi_{h}\right)=\left(E_{3}^{n+1},\varphi_{h}\right). \end{array}$$

Here,

$$\begin{array}{c} \left(E_{1}^{n+1},v_{h}\right)=-\frac{1}{\tau }\int_{t_{n}}^{t_{n+1}}\left(t-t_{n}\right)\left(u_{tt}\left(t\right),v_{h}\right)dt+\frac{1}{\tau }\int_{t_{n}}^{t_{n+1}}\left(t-t_{n}\right)\left(f_{1t}\left(t\right),v_{h}\right)dt\\ +b\left(u\left(t_{n}\right)-u\left(t_{n+1}\right),u\left(t_{n+1}\right),v_{h}\right)+S\left(\left(B\left(t_{n}\right)-B\left(t_{n+1}\right)\right)\times \mathrm{curl}B\left(t_{n+1}\right),v_{h}\right), \end{array}$$

$$\begin{array}{c} \left(E_{2}^{n+1},C_{h}\right)=-\frac{1}{\tau }\int_{t_{n}}^{t_{n+1}}\left(t-t_{n}\right)\left(B_{tt}\left(t\right),C_{h}\right)dt-\left(u\left(t_{n+1}\right)\times \left(B\left(t_{n}\right)-B\left(t_{n+1}\right)\right),\mathrm{curl}C_{h}\right), \end{array}$$

$$\begin{array}{c} \left(E_{3}^{n+1},\varphi_{h}\right)=-\frac{1}{\tau }\int_{t_{n}}^{t_{n+1}}\left(t-t_{n}\right)\left(\theta_{tt}\left(t\right),\varphi_{h}\right)dt+\frac{1}{\tau }\int_{t_{n}}^{t_{n+1}}\left(t-t_{n}\right)\left(f_{2t}\left(t\right),\varphi_{h}\right)dt\\ +b(u\left(t_{n}\right)-u\left(t_{n+1}\right),\theta \left(t_{n+1}\right),\varphi_{h}). \end{array}$$

Using the decomposition and setting $v_{h}=2\tau \Lambda_{u,h}^{n+1}$ in (12), $C_{h}=2\tau \phi_{B,h}^{n+1}$ in (13), $\varphi_{h}=2\tau \phi_{\theta ,h}^{n+1}$ in (14), and adding the three equations, we deduce that

$$\begin{array}{cc} \; & ∥\Lambda_{u,h}^{n+1}{∥}_{0,2}^{2}-∥\phi_{u,h}^{n}{∥}_{0,2}^{2}+∥\Lambda_{u,h}^{n+1}-\phi_{u,h}^{n}{∥}_{0,2}^{2}+2\nu \tau ∥\nabla \Lambda_{u,h}^{n+1}{∥}_{0,2}^{2}+S(∥\phi_{B,h}^{n+1}{∥}_{0,2}^{2}-{∥\phi_{B,h}^{n}∥}_{0,2}^{2}\\ & +∥\phi_{B,h}^{n+1}-\phi_{B,h}^{n}{∥}_{0,2}^{2})+\frac{2\tau S}{R_{m}}∥\mathrm{curl}\phi_{B,h}^{n+1}{∥}_{0,2}^{2}+∥\phi_{\theta ,h}^{n+1}{∥}_{0,2}^{2}-{∥\phi_{\theta ,h}^{n}∥}_{0,2}^{2}\\ \; & +∥\phi_{\theta ,h}^{n+1}-\phi_{\theta ,h}^{n}{∥}_{0,2}^{2}+2\kappa \tau {∥\nabla \phi_{\theta ,h}^{n+1}∥}_{0,2}^{2}\\ \; & =2\tau \left(d_{t}\eta_{u}^{n+1},\Lambda_{u,h}^{n+1}\right)+2\tau S\left(d_{t}\eta_{B}^{n+1},\phi_{B,h}^{n+1}\right)+2\tau \left(d_{t}\eta_{\theta }^{n+1},\phi_{\theta ,h}^{n+1}\right)\\ \; & +2\nu \tau \left(\nabla \eta_{u}^{n+1},\nabla \Lambda_{u,h}^{n+1}\right)+\frac{2\tau S}{R_{m}}\left(\mathrm{curl}\eta_{B}^{n+1},\mathrm{curl}\phi_{B,h}^{n+1}\right)+2\kappa \tau \left(\nabla \eta_{\theta }^{n+1},\nabla \phi_{\theta ,h}^{n+1}\right)\\ \; & +2\tau b\left(u\left(t_{n}\right),u\left(t_{n+1}\right),\Lambda_{u,h}^{n+1}\right)-2\tau b\left(u_{h}^{n},{\hat{u}}_{h}^{n+1},\Lambda_{u,h}^{n+1}\right)+2\tau S\left(B\left(t_{n}\right)\times \mathrm{curl}B\left(t_{n+1}\right),\Lambda_{u,h}^{n+1}\right)\\ \; & -2\tau S\left(B_{h}^{n}\times \mathrm{curl}B_{h}^{n+1},\Lambda_{u,h}^{n+1}\right)-2\tau \beta \left(\eta_{\theta }^{n+1}-\phi_{\theta ,h}^{n+1},\Lambda_{u,h}^{n+1}\right)-2\tau S\left(u\left(t_{n+1}\right)\times B\left(t_{n}\right),\mathrm{curl}\phi_{B,h}^{n+1}\right)\\ \; & +2\tau S\left({\hat{u}}_{h}^{n+1}\times B_{h}^{n},\mathrm{curl}\phi_{B,h}^{n+1}\right)+2\tau b\left(u\left(t_{n}\right),\theta \left(t_{n+1}\right),\phi_{\theta ,h}^{n+1}\right)-2\tau b\left(u_{h}^{n},\theta_{h}^{n+1},\phi_{\theta ,h}^{n+1}\right)\\ \; & -2\tau \left(E_{1}^{n+1},\Lambda_{u,h}^{n+1}\right)-2\tau \left(E_{2}^{n+1},\phi_{B,h}^{n+1}\right)-2\tau \left(E_{3}^{n+1},\phi_{\theta ,h}^{n+1}\right). \end{array}$$

Inserting $\pm 2\tau b\left(u_{h}^{n},u\left(t_{n+1}\right),\Lambda_{u,h}^{n+1}\right),\pm 2\tau b\left({\hat{u}}_{h}^{n+1},\eta_{u}^{n+1},\Lambda_{u,h}^{n+1}\right),\pm 2\tau S\left(B_{h}^{n}\times \mathrm{curl}B\left(t_{n+1}\right),\Lambda_{u,h}^{n+1}\right),\pm 2\tau S\left(u\left(t_{n+1}\right)\times B_{h}^{n},\mathrm{curl}\phi_{B,h}^{n+1}\right),\pm 2\tau S\left(\eta_{u}^{n+1}\times B_{h}^{n+1},\mathrm{curl}\phi_{B,h}^{n+1}\right),\pm 2\tau b\left(u_{h}^{n},\theta \left(t_{n+1}\right),\phi_{\theta ,h}^{n+1}\right),\pm 2\tau b\left({\hat{u}}_{h}^{n+1},\eta_{\theta }^{n+1},\phi_{\theta ,h}^{n+1}\right).$ Then, the above equation can be rewritten as follows:

(15) $$\begin{array}{c} \begin{array}{cc} & ∥\Lambda_{u,h}^{n+1}{∥}_{0,2}^{2}-∥\phi_{u,h}^{n}{∥}_{0,2}^{2}+∥\Lambda_{u,h}^{n+1}-\phi_{u,h}^{n}{∥}_{0,2}^{2}+2\nu \tau ∥\nabla \Lambda_{u,h}^{n+1}{∥}_{0,2}^{2}+S(∥\phi_{B,h}^{n+1}{∥}_{0,2}^{2}-{∥\phi_{B,h}^{n}∥}_{0,2}^{2}\\ & +∥\phi_{B,h}^{n+1}-\phi_{B,h}^{n}{∥}_{0,2}^{2})+\frac{2\tau S}{R_{m}}∥\mathrm{curl}\phi_{B,h}^{n+1}{∥}_{0,2}^{2}+∥\phi_{\theta ,h}^{n+1}{∥}_{0,2}^{2}-{∥\phi_{\theta ,h}^{n}∥}_{0,2}^{2}\\ & +∥\phi_{\theta ,h}^{n+1}-\phi_{\theta ,h}^{n}{∥}_{0,2}^{2}+2\kappa \tau {∥\nabla \phi_{\theta ,h}^{n+1}∥}_{0,2}^{2}\\ & =2\tau \left(d_{t}\eta_{u}^{n+1},\Lambda_{u,h}^{n+1}\right)+2\tau S\left(d_{t}\eta_{B}^{n+1},\phi_{B,h}^{n+1}\right)+2\tau \left(d_{t}\eta_{\theta }^{n+1},\phi_{\theta ,h}^{n+1}\right)\\ & +2\nu \tau \left(\nabla \eta_{u}^{n+1},\nabla \Lambda_{u,h}^{n+1}\right)+\frac{2\tau S}{R_{m}}\left(\mathrm{curl}\eta_{B}^{n+1},\mathrm{curl}\phi_{B,h}^{n+1}\right)+2\kappa \tau \left(\nabla \eta_{\theta }^{n+1},\nabla \phi_{\theta ,h}^{n+1}\right)\\ & +2\tau b\left(\eta_{u}^{n},u\left(t_{n+1}\right),\Lambda_{u,h}^{n+1}\right)-2\tau b\left(\phi_{u,h}^{n},u\left(t_{n+1}\right),\Lambda_{u,h}^{n+1}\right)+2\tau b\left(-\tau d_{t}{\hat{u}}_{h}^{n+1},\eta_{u}^{n+1},\Lambda_{u,h}^{n+1}\right)\\ & +2\tau b\left({\hat{u}}_{h}^{n+1},\eta_{u}^{n+1},\Lambda_{u,h}^{n+1}\right)+2\tau S\left(\eta_{B}^{n}\times \mathrm{curl}B\left(t_{n+1}\right),\Lambda_{u,h}^{n+1}\right)-2\tau S\left(\phi_{B,h}^{n}\times \mathrm{curl}B\left(t_{n+1}\right),\Lambda_{u,h}^{n+1}\right)\\ & +2\tau S\left(B_{h}^{n}\times \mathrm{curl}\eta_{B}^{n+1},\Lambda_{u,h}^{n+1}\right)-2\tau S\left(u\left(t_{n+1}\right)\times \eta_{B}^{n},\mathrm{curl}\phi_{B,h}^{n+1}\right)-2\tau \beta \left(\eta_{\theta }^{n+1}-\phi_{\theta ,h}^{n+1},\Lambda_{u,h}^{n+1}\right)\\ & +2\tau S\left(u\left(t_{n+1}\right)\times \phi_{B,h}^{n},\mathrm{curl}\phi_{B,h}^{n+1}\right)-2\tau S\left(\eta_{u}^{n+1}\times \left(-\tau d_{t}B_{h}^{n+1}\right),\mathrm{curl}\phi_{B,h}^{n+1}\right)\\ & -2\tau S\left(\eta_{u}^{n+1}\times B_{h}^{n+1},\mathrm{curl}\phi_{B,h}^{n+1}\right)+2\tau b\left(\eta_{u}^{n},\theta \left(t_{n+1}\right),\phi_{\theta ,h}^{n+1}\right)\\ & -2\tau b\left(\phi_{u,h}^{n},\theta \left(t_{n+1}\right),\phi_{\theta ,h}^{n+1}\right)+2\tau b\left(-\tau d_{t}{\hat{u}}_{h}^{n+1},\eta_{\theta }^{n+1},\phi_{\theta ,h}^{n+1}\right)\\ & +2\tau b\left({\hat{u}}_{h}^{n+1},\eta_{\theta }^{n+1},\phi_{\theta ,h}^{n+1}\right)-2\tau \left(E_{1}^{n+1},\Lambda_{u,h}^{n+1}\right)-2\tau \left(E_{2}^{n+1},\phi_{B,h}^{n+1}\right)-2\tau \left(E_{3}^{n+1},\phi_{\theta ,h}^{n+1}\right). \end{array} \end{array}$$

**Step 2**: [**The estimation of the right-hand side terms of error equations**] We now estimate each term of the right-hand sides of (15) separately.

$$\begin{array}{cc} 2\tau |(d_{t}\eta_{u}^{n+1},\Lambda_{u,h}^{n+1})| & \leq C\int_{t_{n}}^{t_{n+1}}∥\eta_{ut}{∥}_{0,2}^{2}dt+\frac{\nu \tau }{11}{∥\nabla \Lambda_{u,h}^{n+1}∥}_{0,2}^{2},\\ 2\tau S|(d_{t}\eta_{B}^{n+1},\phi_{B,h}^{n+1})| & \leq C\int_{t_{n}}^{t_{n+1}}∥\eta_{Bt}{∥}_{0,2}^{2}dt+\frac{\tau S}{7R_{m}}{∥\mathrm{curl}\phi_{B,h}^{n+1}∥}_{0,2}^{2},\\ 2\tau |(d_{t}\eta_{\theta }^{n+1},\phi_{\theta ,h}^{n+1})| & \leq \int_{t_{n}}^{t_{n+1}}∥\eta_{\theta t}{∥}_{0,2}^{2}dt+\frac{\kappa \tau }{8}{∥\nabla \phi_{\theta ,h}^{n+1}∥}_{0,2}^{2},\\ 2\nu \tau |(\nabla \eta_{u}^{n+1},\nabla \Lambda_{u,h}^{n+1})| & \leq C\tau ∥\nabla \eta_{u}^{n+1}{∥}_{0,2}^{2}+\frac{\nu \tau }{11}{∥\nabla \Lambda_{u,h}^{n+1}∥}_{0,2}^{2},\\ \frac{2\tau S}{R_{m}}\left|\left(\mathrm{curl}\eta_{B}^{n+1},\mathrm{curl}\phi_{B,h}^{n+1}\right)\right| & \leq C\tau ∥\mathrm{curl}\eta_{B}^{n+1}{∥}_{0,2}^{2}+\frac{\tau S}{7R_{m}}{∥\mathrm{curl}\phi_{B,h}^{n+1}∥}_{0,2}^{2},\\ 2\kappa \tau |(\nabla \eta_{\theta }^{n+1},\nabla \phi_{\theta ,h}^{n+1})| & \leq C\tau ∥\nabla \eta_{\theta }^{n+1}{∥}_{0,2}^{2}+\frac{\kappa \tau }{8}{∥\nabla \phi_{\theta ,h}^{n+1}∥}_{0,2}^{2}. \end{array}$$

Next, using (3), the inverse inequality, the Canchy–Schwarz and Young’s inequalities, we arrive at

$$\begin{array}{cc} 2\tau |b(\eta_{u}^{n},u\left(t_{n+1}\right),\Lambda_{u,h}^{n+1})| & \leq 2\tau ∥\nabla \eta_{u}^{n}{∥}_{0,2}∥\nabla u\left(t_{n+1}\right){∥}_{0,2}{∥\nabla \Lambda_{u,h}^{n+1}∥}_{0,2}\\ \; & \leq C\tau ∥\nabla u\left(t_{n+1}\right){∥}_{0,2}^{2}∥\nabla \eta_{u}^{n}{∥}_{0,2}^{2}+\frac{\nu \tau }{11}{∥\nabla \Lambda_{u,h}^{n+1}∥}_{0,2}^{2},\\ 2\tau |b(\phi_{u,h}^{n},u\left(t_{n+1}\right),\Lambda_{u,h}^{n+1})| & \leq C\tau ∥u\left(t_{n+1}\right){∥}_{2,2}^{2}∥\phi_{u,h}^{n}{∥}_{0,2}^{2}+\frac{\nu \tau }{11}{∥\nabla \Lambda_{u,h}^{n+1}∥}_{0,2}^{2},\\ 2\tau |b(u_{h}^{n}-{\hat{u}}_{h}^{n+1},\eta_{u}^{n+1},\Lambda_{u,h}^{n+1})| & \leq C\tau h^{-1/2}∥u_{h}^{n}-{\hat{u}}_{h}^{n+1}{∥}_{0,2}∥\nabla \eta_{u}^{n+1}{∥}_{0,2}{∥\nabla \Lambda_{u,h}^{n+1}∥}_{0,2}\\ \; & \leq C\tau h^{-1}∥u_{h}^{n}-{\hat{u}}_{h}^{n+1}{∥}_{0,2}^{2}∥\nabla \eta_{u}^{n+1}{∥}_{0,2}^{2}+\frac{\nu \tau }{11}{∥\nabla \Lambda_{u,h}^{n+1}∥}_{0,2}^{2},\\ 2\tau |b({\hat{u}}_{h}^{n+1},\eta_{u}^{n+1},\Lambda_{u,h}^{n+1})| & \leq C\tau ∥\nabla {\hat{u}}_{h}^{n+1}{∥}_{0,2}^{2}∥\nabla \eta_{u}^{n+1}{∥}_{0,2}^{2}+\frac{\nu \tau }{11}{∥\nabla \Lambda_{u,h}^{n+1}∥}_{0,2}^{2}. \end{array}$$

Then, using (4) with Cauchy–Schwarz and Young’s inequalities, we obtain

$$\begin{array}{cc} 2\tau S|(\eta_{B}^{n}\times \mathrm{curl}B\left(t_{n+1}\right),\Lambda_{u,h}^{n+1})| & \leq C\tau ∥B\left(t_{n+1}\right){∥}_{2,2}^{2}∥\eta_{B}^{n}{∥}_{0,2}^{2}+\frac{\nu \tau }{11}{∥\nabla \Lambda_{u,h}^{n+1}∥}_{0,2}^{2},\\ 2\tau S|(\phi_{B,h}^{n}\times \mathrm{curl}B\left(t_{n+1}\right),\Lambda_{u,h}^{n+1})| & \leq C\tau ∥B\left(t_{n+1}\right){∥}_{2,2}^{2}∥\phi_{B,h}^{n}{∥}_{0,2}^{2}+\frac{\nu \tau }{11}{∥\nabla \Lambda_{u,h}^{n+1}∥}_{0,2}^{2},\\ 2\tau S|(B_{h}^{n}\times \mathrm{curl}\eta_{B}^{n+1},\Lambda_{u,h}^{n+1})| & \leq C\tau ∥\mathrm{curl}B_{h}^{n}{∥}_{0,2}^{2}∥\mathrm{curl}\eta_{B}^{n+1}{∥}_{0,2}^{2}+\frac{\nu \tau }{11}{∥\nabla \Lambda_{u,h}^{n+1}∥}_{0,2}^{2}. \end{array}$$

Similarly, we obtain

$$\begin{array}{cc} 2\tau S|(u\left(t_{n+1}\right)\times \eta_{B}^{n},\mathrm{curl}\phi_{B,h}^{n+1})| & \leq C\tau ∥u\left(t_{n+1}\right){∥}_{2,2}^{2}∥\eta_{B}^{n}{∥}_{0,2}^{2}+\frac{\tau S}{7R_{m}}{∥\mathrm{curl}\phi_{B,h}^{n+1}∥}_{0,2}^{2},\\ 2\tau S|(u\left(t_{n+1}\right)\times \phi_{B,h}^{n},\mathrm{curl}\phi_{B,h}^{n+1})| & \leq C\tau ∥u\left(t_{n+1}\right){∥}_{2,2}^{2}∥\phi_{B,h}^{n}{∥}_{0,2}^{2}+\frac{\tau S}{7R_{m}}{∥\mathrm{curl}\phi_{B,h}^{n+1}∥}_{0,2}^{2},\\ 2\tau S|(\eta_{u}^{n+1}\times \left(-\tau d_{t}B_{h}^{n+1}\right),\mathrm{curl}\phi_{B,h}^{n+1})| & \leq C\tau ∥B_{h}^{n}-B_{h}^{n+1}{∥}_{0,2}^{2}h^{-1}∥\nabla \eta_{u}^{n+1}{∥}_{0,2}^{2}+\frac{\tau S}{7R_{m}}{∥\mathrm{curl}\phi_{B,h}^{n+1}∥}_{0,2}^{2},\\ 2\tau S|(\eta_{u}^{n+1}\times B_{h}^{n+1},\mathrm{curl}\phi_{B,h}^{n+1})| & \leq C\tau ∥\mathrm{curl}B_{h}^{n+1}{∥}_{0,2}^{2}∥\nabla \eta_{u}^{n+1}{∥}_{0,2}^{2}+\frac{\tau S}{7R_{m}}{∥\mathrm{curl}\phi_{B,h}^{n+1}∥}_{0,2}^{2}. \end{array}$$

Showing that

$$\begin{array}{cc} 2\tau |b(\eta_{u}^{n},\theta \left(t_{n+1}\right),\phi_{\theta ,h}^{n+1})| & \leq C\tau ∥\nabla \eta_{u}^{n}{∥}_{0,2}^{2}∥\nabla \theta \left(t_{n+1}\right){∥}_{0,2}^{2}+\frac{\kappa \tau }{8}{∥\nabla \phi_{\theta ,h}^{n+1}∥}_{0,2}^{2},\\ 2\tau |b(\phi_{u,h}^{n},\theta \left(t_{n+1}\right),\phi_{\theta ,h}^{n+1})| & \leq C\tau ∥\theta \left(t_{n+1}\right){∥}_{2,2}^{2}∥\phi_{u,h}^{n}{∥}_{0,2}^{2}+\frac{\kappa \tau }{8}{∥\nabla \phi_{\theta ,h}^{n+1}∥}_{0,2}^{2},\\ 2\tau |b(-\tau d_{t}{\hat{u}}_{h}^{n+1},\eta_{\theta }^{n+1},\phi_{\theta ,h}^{n+1})| & \leq C\tau h^{-1}∥u_{h}^{n}-{\hat{u}}_{h}^{n+1}{∥}_{0,2}^{2}∥\nabla \eta_{\theta }^{n+1}{∥}_{0,2}^{2}+\frac{\kappa \tau }{8}{∥\nabla \phi_{\theta ,h}^{n+1}∥}_{0,2}^{2},\\ 2\tau |b({\hat{u}}_{h}^{n+1},\eta_{\theta }^{n+1},\phi_{\theta ,h}^{n+1})| & \leq C\tau ∥\nabla {\hat{u}}_{h}^{n+1}{∥}_{0,2}^{2}∥\nabla \eta_{\theta }^{n+1}{∥}_{0,2}^{2}+\frac{\kappa \tau }{8}{∥\nabla \phi_{\theta ,h}^{n+1}∥}_{0,2}^{2},\\ 2\tau |\beta (\eta_{\theta }^{n+1}-\phi_{\theta ,h}^{n+1},\Lambda_{u,h}^{n+1})| & \leq C\tau ∥\nabla \eta_{\theta }^{n+1}{∥}_{0,2}^{2}+\frac{\kappa \tau }{8}∥\nabla \phi_{\theta ,h}^{n+1}{∥}_{0,2}^{2}+\frac{\nu \tau }{11}{∥\nabla \Lambda_{u,h}^{n+1}∥}_{0,2}^{2}. \end{array}$$

Further, we can obtain

$$\begin{array}{cc} ∥E_{1}^{n+1}{∥}_{-1,2}^{2} & ={\left(\underset{v_{h}\in X_{h},v_{h}\neq 0}{\sup}\frac{\left|(E_{1}^{n+1},v_{h})\right|}{∥\nabla v_{h}{∥}_{0,2}}\right)}^{2}\\ \; & \leq C\tau (\int_{t_{n}}^{t_{n+1}}(∥f_{1t}{\left(t\right)∥}_{0,2}^{2}+∥u_{tt}{∥}_{-1,2}^{2})dt+∥\nabla u\left(t_{n+1}\right){∥}_{0,2}^{2}\int_{t_{n}}^{t_{n+1}}{∥\nabla u_{t}\left(t\right)∥}_{0,2}^{2}dt\\ \; & +∥\nabla B\left(t_{n+1}\right){∥}_{0,2}^{2}\int_{t_{n}}^{t_{n+1}}∥\nabla B_{t}\left(t\right){∥}_{0,2}^{2}dt), \end{array}$$

$$\begin{array}{cc} ∥E_{2}^{n+1}{∥}_{-1,2}^{2} & ={\left(\underset{C_{h}\in M_{h},C_{h}\neq 0}{\sup}\frac{\left|(E_{2}^{n+1},C_{h})\right|}{∥\mathrm{curl}C_{h}{∥}_{0,2}}\right)}^{2}\\ \; & \leq C\tau (\int_{t_{n}}^{t_{n+1}}∥B_{tt}{∥}_{-1,2}^{2}dt+∥\nabla u\left(t_{n+1}\right){∥}_{0,2}^{2}\int_{t_{n}}^{t_{n+1}}∥\nabla B_{t}\left(t\right){∥}_{0,2}^{2}dt), \end{array}$$

$$\begin{array}{cc} ∥E_{3}^{n+1}{∥}_{-1,2}^{2} & ={\left(\underset{\varphi_{h}\in W_{h},\varphi_{h}\neq 0}{\sup}\frac{\left|(E_{3}^{n+1},\varphi_{h})\right|}{∥\nabla \varphi_{h}{∥}_{0,2}}\right)}^{2}\\ \; & \leq C\tau (\int_{t_{n}}^{t_{n+1}}(∥f_{2t}{\left(t\right)∥}_{0,2}^{2}+∥\theta_{tt}{∥}_{-1,2}^{2})dt+∥\nabla \theta \left(t_{n+1}\right){∥}_{0,2}^{2}\int_{t_{n}}^{t_{n+1}}∥\nabla u_{t}\left(t\right){∥}_{0,2}^{2}dt). \end{array}$$

For the above three inequalities, we sum n=0 to N−1

$$\begin{array}{cc} \; & \sum_{n=0}^{N-1}(∥E_{1}^{n+1}{∥}_{-1,2}^{2}+∥E_{2}^{n+1}{∥}_{-1,2}^{2}+∥E_{3}^{n+1}{∥}_{-1,2}^{2})\\ \; & \leq C\tau \int_{0}^{T}(∥f_{1t}{\left(t\right)∥}_{0,2}^{2}+∥f_{2t}{\left(t\right)∥}_{0,2}^{2}+∥u_{tt}{∥}_{-1,2}^{2}+∥B_{tt}{∥}_{-1,2}^{2}+∥\theta_{tt}{∥}_{-1,2}^{2})dt\\ \; & +C\tau (∥\nabla u\left(t_{n+1}\right){∥}_{0,2}^{2}+∥\nabla B\left(t_{n+1}\right){∥}_{0,2}^{2}+∥\nabla \theta \left(t_{n+1}\right){∥}_{0,2}^{2})\int_{0}^{T}(∥\nabla u_{t}\left(t\right){∥}_{0,2}^{2}+∥\nabla B_{t}\left(t\right){∥}_{0,2}^{2})dt. \end{array}$$

Based on Assumptions 1 and 3, we arrive at

$$\begin{array}{c} \tau \sum_{n=0}^{N-1}(∥E_{1}^{n+1}{∥}_{-1,2}^{2}+∥E_{2}^{n+1}{∥}_{-1,2}^{2}+∥E_{3}^{n+1}{∥}_{-1,2}^{2})\leq C\tau^{2}. \end{array}$$

**Step 3**: [**The completion of the proof**] Substituting all the above inequalities into the right-hand term of (15) and applying Assumptions 1–3, asserting Lemma 3, yields

$$\begin{array}{cc} \; & ∥\phi_{u,h}^{n+1}{∥}_{0,2}^{2}-∥\phi_{u,h}^{n}{∥}_{0,2}^{2}+∥\Lambda_{u,h}^{n+1}-\phi_{u,h}^{n}{∥}_{0,2}^{2}+∥\Lambda_{u,h}^{n+1}-\phi_{u,h}^{n+1}{∥}_{0,2}^{2}+\nu \tau {∥\nabla \Lambda_{u,h}^{n+1}∥}_{0,2}^{2}\\ \; & +S(∥\phi_{B,h}^{n+1}{∥}_{0,2}^{2}-∥\phi_{B,h}^{n}{∥}_{0,2}^{2}+∥\phi_{B,h}^{n+1}-\phi_{B,h}^{n}{∥}_{0,2}^{2})+\frac{\tau S}{R_{m}}∥\mathrm{curl}\phi_{B,h}^{n+1}{∥}_{0,2}^{2}+∥\phi_{\theta ,h}^{n+1}{∥}_{0,2}^{2}-{∥\phi_{\theta ,h}^{n}∥}_{0,2}^{2}\\ & +∥\phi_{\theta ,h}^{n+1}-\phi_{\theta ,h}^{n}{∥}_{0,2}^{2}+\kappa \tau {∥\nabla \phi_{\theta ,h}^{n+1}∥}_{0,2}^{2}\\ \; & +\beta_{0}(∥\nabla \cdot \phi_{u,h}^{n+1}{∥}_{0,2}^{2}-∥\nabla \cdot \phi_{u,h}^{n}{∥}_{0,2}^{2})+\frac{\beta_{0}}{2}∥\nabla \cdot \left(\phi_{u,h}^{n+1}-\phi_{u,h}^{n}\right){∥}_{0,2}^{2}+\gamma_{0}\tau {∥\nabla \cdot \phi_{u}^{n+1}∥}_{0,2}^{2}\\ \; & \leq \beta_{0}\left(1+2\tau \right)\int_{t_{n}}^{t_{n+1}}∥\nabla \eta_{ut}{∥}_{0,2}^{2}dt+\beta_{0}\tau ∥\nabla \cdot \phi_{u,h}^{n}{∥}_{0,2}^{2}+\gamma_{0}\tau {∥\nabla \eta_{u}^{n+1}∥}_{0,2}^{2}\\ \; & +C(\int_{t_{n}}^{t_{n+1}}∥\eta_{ut}{∥}_{0,2}^{2}dt+\int_{t_{n}}^{t_{n+1}}∥\eta_{Bt}{∥}_{0,2}^{2}dt+\int_{t_{n}}^{t_{n+1}}∥\eta_{\theta t}{∥}_{0,2}^{2}dt+\tau ∥\nabla \eta_{u}^{n+1}{∥}_{0,2}^{2}+\tau ∥\mathrm{curl}\eta_{B}^{n+1}{∥}_{0,2}^{2}\\ & +\tau ∥\nabla \eta_{\theta }^{n+1}{∥}_{0,2}^{2})+C\tau (∥\nabla \eta_{u}^{n}{∥}_{0,2}^{2}+∥\nabla \eta_{B}^{n}{∥}_{0,2}^{2}+∥\phi_{u,h}^{n}{∥}_{0,2}^{2}+S∥\phi_{B,h}^{n}{∥}_{0,2}^{2})\\ \; & +C\tau (h^{-1}∥u_{h}^{n}-{\hat{u}}_{h}^{n+1}{∥}_{0,2}^{2}∥\nabla \eta_{u}^{n+1}{∥}_{0,2}^{2}+h^{-1}∥B_{h}^{n}-B_{h}^{n+1}{∥}_{0,2}^{2}∥\nabla \eta_{u}^{n+1}{∥}_{0,2}^{2}\\ \; & +h^{-1}∥u_{h}^{n}-{\hat{u}}_{h}^{n+1}{∥}_{0,2}^{2}∥\nabla \eta_{\theta }^{n+1}{∥}_{0,2}^{2})+C\tau (∥\nabla {\hat{u}}_{h}^{n+1}{∥}_{0,2}^{2}∥\nabla \eta_{u}^{n+1}{∥}_{0,2}^{2}+∥\mathrm{curl}B_{h}^{n}{∥}_{0,2}^{2}{∥\mathrm{curl}\eta_{B}^{n+1}∥}_{0,2}^{2}\\ \; & +∥\mathrm{curl}B_{h}^{n+1}{∥}_{0,2}^{2}∥\nabla \eta_{u}^{n+1}{∥}_{0,2}^{2}+∥\nabla {\hat{u}}_{h}^{n+1}{∥}_{0,2}^{2}∥\nabla \eta_{\theta }^{n+1}{∥}_{0,2}^{2})\\ \; & +C\tau (∥\nabla \eta_{\theta }^{n+1}{∥}_{0,2}^{2}+∥E_{1}^{n+1}{∥}_{-1,2}^{2}+∥E_{2}^{n+1}{∥}_{-1,2}^{2}+∥E_{3}^{n+1}{∥}_{-1,2}^{2}). \end{array}$$

Then, we sum over time steps and use the results of Lemma 2 and regularity Assumptions 1–3, and arrive at

$$\begin{array}{cc} \; & ∥\phi_{u,h}^{N}{∥}_{0,2}^{2}+\beta_{0}∥\nabla \cdot \phi_{u,h}^{N}{∥}_{0,2}^{2}+S∥\phi_{B,h}^{N}{∥}_{0,2}^{2}+∥\phi_{\theta ,h}^{N}{∥}_{0,2}^{2})\\ \; & +\tau \sum_{n=0}^{N-1}(\nu ∥\nabla \Lambda_{u,h}^{n+1}{∥}_{0,2}^{2}+\frac{S}{R_{m}}∥\mathrm{curl}\phi_{B,h}^{n+1}{∥}_{0,2}^{2}+\kappa ∥\nabla \phi_{\theta ,h}^{n+1}{∥}_{0,2}^{2}+\gamma_{0}∥\nabla \cdot \phi_{u,h}^{n+1}{∥}_{0,2}^{2})\\ \; & \leq C\tau \sum_{n=0}^{N-1}(∥\phi_{u,h}^{n}{∥}_{0,2}^{2}+\beta_{0}∥\nabla \cdot \phi_{u,h}^{n}{∥}_{0,2}^{2}+S∥\phi_{B,h}^{n}{∥}_{0,2}^{2}+∥\phi_{\theta ,h}^{n}{∥}_{0,2}^{2})\\ \; & +C(\tau h^{2}+\tau h+h^{2}+\tau^{2}+∥\phi_{u,h}^{0}{∥}_{0,2}^{2}+\beta_{0}∥\nabla \cdot \phi_{u,h}^{0}{∥}_{0,2}^{2}+S∥\phi_{B,h}^{0}{∥}_{0,2}^{2}+∥\phi_{\theta ,h}^{0}{∥}_{0,2}^{2}). \end{array}$$

Further, we apply Gronwall’s lemma and use $\phi_{u,h}^{0}=\phi_{B,h}^{0}=0,\phi_{\theta ,h}^{0}=0,$ to obtain

$$\begin{array}{cc} \; & ∥\phi_{u,h}^{N}{∥}_{0,2}^{2}+\beta_{0}∥\nabla \cdot \phi_{u,h}^{N}{∥}_{0,2}^{2}+S∥\phi_{B,h}^{N}{∥}_{0,2}^{2}+∥\phi_{\theta ,h}^{N}{∥}_{0,2}^{2})\\ \; & +\tau \sum_{n=0}^{N-1}(\nu ∥\nabla \Lambda_{u,h}^{n+1}{∥}_{0,2}^{2}+\frac{S}{R_{m}}∥\mathrm{curl}\phi_{B,h}^{n+1}{∥}_{0,2}^{2}+\kappa ∥\nabla \phi_{\theta ,h}^{n+1}{∥}_{0,2}^{2}+\gamma_{0}∥\nabla \cdot \phi_{u,h}^{n+1}{∥}_{0,2}^{2})\\ \; & \leq C(\tau h^{2}+\tau h+h^{2}+\tau^{2}). \end{array}$$

Finally, applying the triangle inequality yields the desired results. □

## 6. Numerical Experiment

In this section, we give two numerical experiments to illustrate the reliability of the thermally coupled MHD problem. One aspect is to verify the predicted stability and convergence rates of the previous section, another is to consider the flexibility of large Reynolds number and grad-div stabilization parameters. In the following tests, we use the finite-element pair $\left(P_{1}^{b},P_{1},P_{1},P_{1}\right)$ for the velocity/pressure/magnetic/temperature, respectively.

### 6.1. An Exact Solution Problem

First, we consider an exact solution problem to verify the stability and the convergence rates of Algorithm 1 for problems (1). Let the domain $\Omega =\left[0,1\right]\times \left[0,1\right]\in R^{2}$ and the mesh is obtained by dividing Ω into squares and drawing a diagonal in each square. Therefore, the exact solution (u,p,B,θ) is given by

$$\left\{\begin{array}{c} u_{1}\left(x,y,t\right)=x^{2}{\left(x-1\right)}^{2}y\left(y-1\right)\left(2y-1\right)\cos\left(t\right),\\ u_{2}\left(x,y,t\right)=-x\left(x-1\right)\left(2x-1\right)y^{2}{\left(y-1\right)}^{2}\cos\left(t\right),\\ B_{1}\left(x,y,t\right)=\sin\left(\pi x\right)\cos\left(\pi y\right)\cos\left(t\right),\\ B_{2}\left(x,y,t\right)=-\sin\left(\pi y\right)\cos\left(\pi x\right)\cos\left(t\right),\\ p(x,y,t)=(2x-1)(2y-1)\cos(t),\\ \theta \left(x,y,t\right)=u_{1}+u_{2}, \end{array}\right.\;$$

then, the external force terms $f_{1}$ and $f_{2}$, boundary conditions, and initial values in Equations (1) are selected to correspond to the exact solution. The parameters $\kappa =S=R_{m}=1$, end time T=1.

In Table 1, we set $\beta_{0}=0.2,\gamma_{0}=1,\nu =1$ for convergence rates and vary the mesh size 1/h between 4, 8, 16, 32, 64. The expected accuracy is consistent with theoretical results. In addition, we can get the $H^{1}\left(\Omega \right)$-norm convergence rates of velocity, magnetic, and temperature fields to be O(h), the $L^{2}\left(\Omega \right)$-norm convergence rates of velocity magnetic, and temperature fields are $O(h^{2})$.

Next, our test is for Re increasing. We fix τ=h,h=1/32 and set $\beta_{0}=0.2,\gamma_{0}=1.$ In Table 2, errors for velocity and pressure with increasing Re of the method without grad-div and modular grad-div stabilization method are compared. The corresponding solutions are no-Stab and Modular. We observe that the error of the proposed algorithm hardly increases. However, the approximate solutions generated by the no-Stab method is getting worse and worse, especially for gradient and divergence results of velocity.

Finally, we fix Re=1,τ=h,h=1/32, vary the grad-div parameters $0.1\leq \beta_{0}\leq$$10^{5}$ and $0.1\leq \gamma_{0}\leq$$10^{5}$. The results are presented in Table 3. We observe that the result of velocity divergence errors of our method becomes small as $\gamma_{0}$ increases, but $\beta_{0}$ has not much impact on them.

### 6.2. Thermally Driven Cavity Flow Problem

In this experiment, we consider thermal driven cavity flow [36] in 2D to verify the efficiency of the proposed algorithm with high Reynolds number. The computation domain is Ω=[0,1]×[0,1] and we set the external force terms $f_{1}=0,f_{2}=0$. The initial conditions are given by $B^{0}=0,u^{0}=0$ and $\theta^{0}=0$. Moreover, the boundary conditions are given as follows:

$$\left\{\begin{array}{c} u=0\;\mathrm{on}\;\partial \Omega ,\\ B\times n=(1,0)\times n\;\mathrm{on}\;\partial \Omega ,\\ \frac{\partial \theta }{\partial n}=0\;\;\mathrm{on}\mathrm{the}\mathrm{top}\mathrm{and}\mathrm{bottom}\mathrm{wall},\\ \theta =1\;\;\mathrm{on}\mathrm{the}\mathrm{left}\mathrm{wall}\mathrm{and}\;\;\theta =0\;\;\mathrm{on}\mathrm{the}\mathrm{right}\mathrm{wall}. \end{array}\right.$$

**Figure 1:**
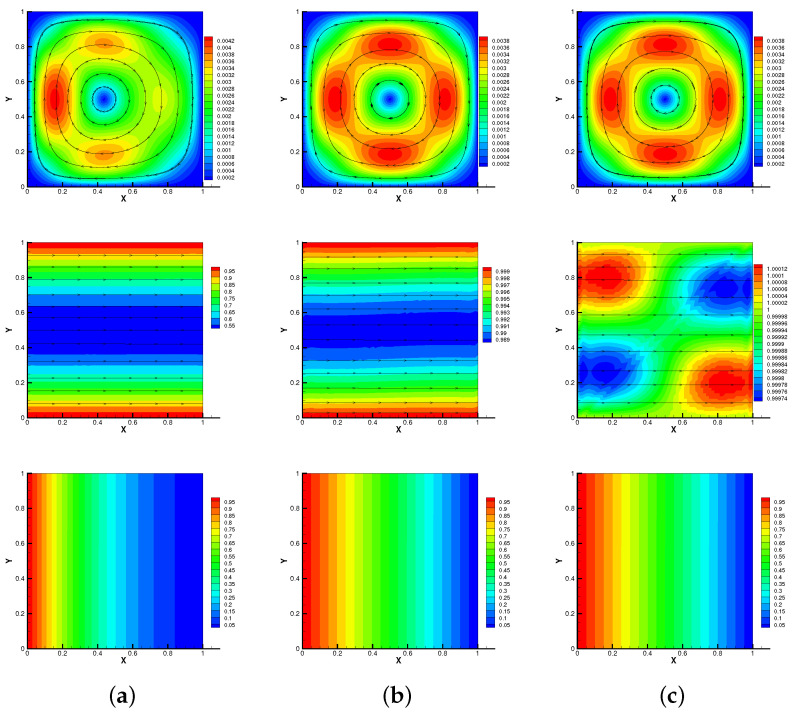
No-Stab method: streamlines of velocity and magnetic, isotherms of temperature for T=0.1 s (**a**), 0.5 s (**b**), 1 s (**c**) with Reynolds number Re=1.

**Figure 2:**
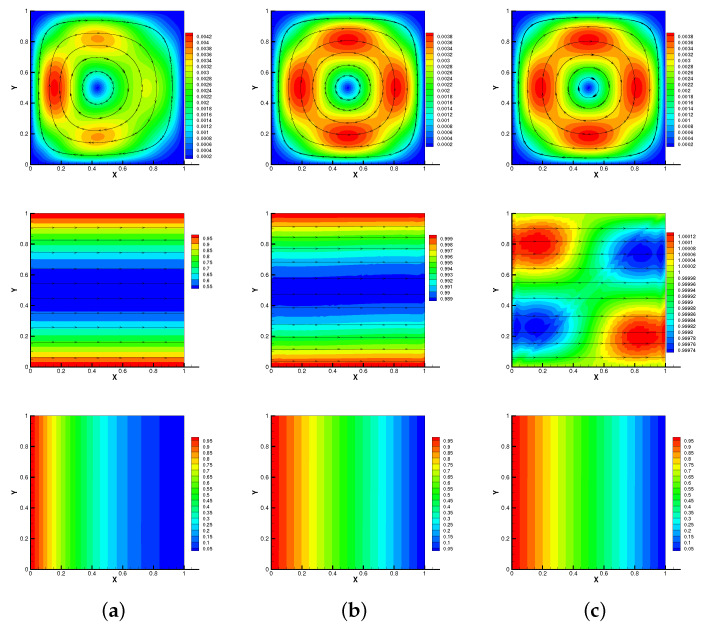
Modular grad-div stabilization method: streamlines of velocity and magnetic, isotherms of temperature for T=0.1 s (**a**), 0.5 s (**b**), 1 s (**c**) with Reynolds number Re=1.

Here, we set $h=1/64,\tau =0.01,S=R_{m}=\kappa =\beta_{0}=\gamma_{0}=1.$ As shown in Figure 1 and Figure 2, we plot streamlines of velocity and magnetic, isotherms of temperature of methods no-Stab and our proposed method at different times when the Reynolds number is Re=1. We can observe that the results of our proposed method are consistent with no-Stab method, which verifies its correctness.

**Figure 3:**
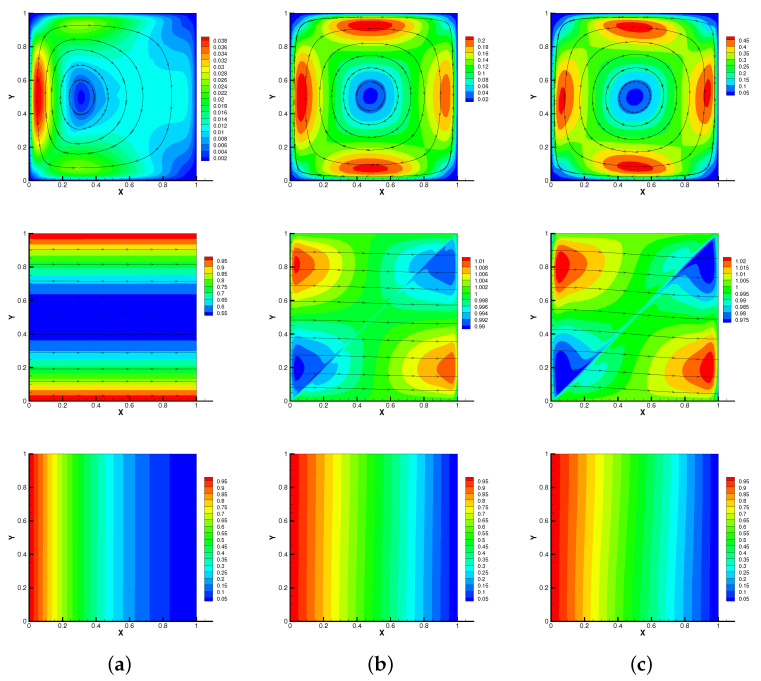
Modular grad-div stabilization method: streamlines of velocity and magnetic, isotherms of temperature for T=0.1 s (**a**), 1 s (**b**), 6 s (**c**) with Reynolds number Re=$10^{6}$.

Moreover, in Figure 3, we show streamlines of velocity and magnetic, isotherms of temperature of our method at different times when the Reynolds number is Re=$10^{6}$. As time goes on, the vortex of the velocity streamline gradually moves from left to right, the streamline of the magnetic field gradually becomes curved in Figure 3 (Re=$10^{6}$). At the same time, due to the temperature difference between the left and right walls, the isotherm also gradually becomes curved. However, these results do not change significantly when Re=1, as can be seen from Figure 1 and Figure 2. It is worth noting that the no-Stab method is divergent with Re=$10^{6}$ when T=3 s, but the modular grad-div stabilization method is still convergent in this case. So, we can find that the proposed method is efficient for the thermally driven cavity flow problem with relatively large Reynolds numbers.

## 7. Conclusions

We developed a first-order fully discrete modular grad-div stabilization algorithm for time-dependent thermally coupled MHD equations. The advantages of this scheme is to keep the conservation of mass as much as possible and its effectiveness with high Reynolds number and large grad-div stabilization parameters. Then, the scheme is proven to be stable and convergent. When compared without grad-div stabilization solutions, our algorithm exhibits a smaller divergence error of the velocity and shows how $\beta_{0}$ and $\gamma_{0}$ influence this effect. Moreover, we also confirm that the scheme still maintains the advantage with high Re and grad-div stabilization parameters. In the future, we will consider high-order schemes with modular grad-div stabilization.

## Figures and Tables

**Table 1:** Errors and convergence rates of the considered scheme with $\tau =h^{2}$ at T=1.

1/h	$∥\nabla \left(u-u_{h}^{n+1}\right){∥}_{0}$	Rate	$∥u-u_{h}^{n+1}{∥}_{0}$	Rate	$∥p-p_{h}^{n+1}{∥}_{0}$	Rate	$∥\nabla {\left(B-B_{h}\right)}^{n+1}{∥}_{0,2}$	Rate
4	$1.06\times 10^{-2}$	-	$1.22\times 10^{-3}$	-	$1.43\times 10^{-2}$	-	$6.49\times 10^{-1}$	-
8	$5.36\times 10^{-3}$	0.98	$3.62\times 10^{-4}$	1.75	$4.35\times 10^{-3}$	1.72	$3.31\times 10^{-1}$	0.97
16	$2.66\times 10^{-3}$	1.01	$8.65\times 10^{-5}$	2.06	$1.34\times 10^{-3}$	1.69	$1.66\times 10^{-1}$	0.99
32	$1.33\times 10^{-3}$	1.00	$2.09\times 10^{-5}$	2.05	$4.23\times 10^{-4}$	1.66	$8.33\times 10^{-2}$	1.00
64	$6.67\times 10^{-3}$	1.01	$5.25\times 10^{-6}$	2.01	$1.88\times 10^{-4}$	1.55	$4.17\times 10^{-2}$	1.00
1/h	$∥B-B_{h}^{n+1}{∥}_{0}$	**Rate**	$∥\nabla \left(T-T_{h}^{n+1}\right){∥}_{0}$	**Rate**	$∥T-T_{h}^{n+1}{∥}_{0}$	**Rate**		
4	$5.44\times 10^{-2}$	–	$7.93\times 10^{-3}$	–	$6.14\times 10^{-4}$	–		
8	$1.51\times 10^{-2}$	1.85	$4.11\times 10^{-3}$	0.95	$1.63\times 10^{-4}$	1.91		
16	$3.87\times 10^{-3}$	1.96	$2.08\times 10^{-3}$	0.98	$4.17\times 10^{-5}$	1.97		
32	$9.69\times 10^{-5}$	2.00	$1.04\times 10^{-3}$	1.00	$1.05\times 10^{-5}$	1.99		
64	$2.42\times 10^{-4}$	2.00	$5.21\times 10^{-4}$	1.00	$2.62\times 10^{-6}$	2.00		

**Table 2:** Errors for velocity and pressure with increasing Re.

Parameter	$∥|u_{h}-u{∥|}_{\infty ,0}$	-	$∥|\nabla \cdot \left(u_{h}-u\right){∥|}_{0,2}$	-	$∥|\nabla \left(u_{h}-u\right){∥|}_{0,2}$	-	$∥|p_{h}-p{∥|}_{0,2}$	-
1/Re	**No-Stab**	**Modular**	**No-Stab**	**Modular**	**No-Stab**	**Modular**	**No-Stab**	**Modular**
1	$3.15\times 10^{-5}$	$6.15\times 10^{-5}$	$1.06\times 10^{-3}$	$1.12\times 10^{-3}$	$2.00\times 10^{-3}$	$2.18\times 10^{-3}$	$2.09\times 10^{-3}$	$2.90\times 10^{-3}$
$10^{1}$	$3.79\times 10^{-5}$	$1.24\times 10^{-4}$	$1.83\times 10^{-3}$	$1.10\times 10^{-3}$	$3.04\times 10^{-3}$	$2.29\times 10^{-3}$	$2.85\times 10^{-3}$	$2.85\times 10^{-3}$
$10^{2}$	$1.19\times 10^{-4}$	$3.07\times 10^{-4}$	$1.47\times 10^{-2}$	$1.05\times 10^{-3}$	$2.30\times 10^{-2}$	$3.04\times 10^{-3}$	$2.85\times 10^{-3}$	$2.85\times 10^{-3}$
$10^{3}$	$1.16\times 10^{-3}$	$4.04\times 10^{-4}$	$1.43\times 10^{-1}$	$1.04\times 10^{-3}$	$2.27\times 10^{-1}$	$3.62\times 10^{-3}$	$2.85\times 10^{-3}$	$2.85\times 10^{-3}$
$10^{4}$	$9.95\times 10^{-3}$	$4.18\times 10^{-4}$	1.26	$1.05\times 10^{-3}$	2.00	$3.75\times 10^{-3}$	$2.86\times 10^{-3}$	$2.85\times 10^{-3}$
$10^{5}$	$3.92\times 10^{-2}$	$4.19\times 10^{-4}$	4.13	$1.05\times 10^{-3}$	6.56	$3.77\times 10^{-3}$	$2.97\times 10^{-3}$	$2.85\times 10^{-3}$
$10^{6}$	5.17e-02	$4.20\times 10^{-4}$	5.02	$1.05\times 10^{-3}$	7.98	$3.77\times 10^{-3}$	$3.04\times 10^{-3}$	$2.85\times 10^{-3}$

**Table 3:** Velocity errors and divergence of the modular grad-div methods with different $\beta_{0},\gamma_{0}$.

$\beta_{0}$	$\gamma_{0}$	$∥|\nabla \left(u_{h}-u\right){∥|}_{2,0}$	$∥|\nabla \cdot \left(u_{h}-u\right){∥|}_{2,0}$	$∥\nabla \cdot u_{h}^{N}{∥}_{2,0}$	$\beta_{0}$	$\gamma_{0}$	$∥|\nabla \left(u_{h}-u\right){∥|}_{2,0}$	$∥|\nabla \cdot \left(u_{h}-u\right){∥|}_{2,0}$	$∥\nabla \cdot u_{h}^{N}{∥}_{2,0}$
0	0.1	$2.10\times 10^{-3}$	$1.00\times 10^{-3}$	$6.40\times 10^{-4}$	0.1	1	$2.15\times 10^{-3}$	$1.05\times 10^{-3}$	$6.30\times 10^{-4}$
0	1	$2.16\times 10^{-3}$	$9.85\times 10^{-4}$	$6.28\times 10^{-4}$	1	1	$2.63\times 10^{-3}$	$1.55\times 10^{-3}$	$9.33\times 10^{-4}$
0	10	$3.28\times 10^{-3}$	$8.79\times 10^{-4}$	$5.59\times 10^{-4}$	10	1	$3.55\times 10^{-3}$	$2.07\times 10^{-3}$	$1.89\times 10^{-3}$
0	100	$9.72\times 10^{-2}$	$5.43\times 10^{-4}$	$4.41\times 10^{-4}$	100	1	$5.07\times 10^{-3}$	$2.18\times 10^{-3}$	$2.12\times 10^{-3}$
0	1000	$1.97\times 10^{-2}$	$1.47\times 10^{-4}$	$9.03\times 10^{-5}$	1000	1	$6.79\times 10^{-3}$	$2.21\times 10^{-3}$	$2.20\times 10^{-3}$
0	10,000	$2.36\times 10^{-2}$	$1.85\times 10^{-5}$	$1.12\times 10^{-5}$	10,000	1	$7.16\times 10^{-3}$	$2.22\times 10^{-3}$	$2.22\times 10^{-3}$
0	100,000	$2.41\times 10^{-2}$	$1.90\times 10^{-6}$	$1.15\times 10^{-6}$	100,000	1	$7.20\times 10^{-3}$	$2.22\times 10^{-3}$	$2.22\times 10^{-3}$

## Data Availability

Not applicable.
